# Exploring access to government-led support for children with disabilities in Bangladesh

**DOI:** 10.1371/journal.pone.0235439

**Published:** 2020-07-02

**Authors:** Reshma Parvin Nuri, Setareh Ghahari, Heather Michelle Aldersey, Ahmed Shafiqul Huque

**Affiliations:** 1 School of Rehabilitation Therapy, Queen’s University, Kingston, Ontario, Canada; 2 Department of Political Science, McMaster University, Hamilton, Ontario, Canada; Università degli Studi di Perugia, ITALY

## Abstract

While access to support for individuals with disabilities has attracted international attention, children with disabilities and their families continue to face a range of barriers that limit their timely access to the needed support, including health service. This is even worse for children with disabilities living in resource poor settings like Bangladesh. The objective of this study was to determine the extent to which families of children with disabilities have knowledge about and access to government support for their children with disabilities in Bangladesh. We employed a cross-sectional study among 393 families of children with disabilities who sought services from the Centre for the Rehabilitation of the Paralysed for their children with disabilities in Bangladesh. We used chi-square test to measure the association between categorical variables and, Mann-Whitney U-test to compare mean across different sub-groups. Overall, family members of children with disabilities have limited knowledge about and access to government support. We found a significant association between knowledge and access to government support (p<0.001). Family members with children with disabilities aged younger than six years had less access to government support (p<0.001). We thus concluded with an urgent call on government agencies and service providers to provide relevant and timely information to families of children with disabilities to enable them to access the needed support.

## Introduction

It is estimated that about 15% of the global population lives with some form of disability and 80% of them are living in low- and middle-income countries (LMICs) [1,2]. According to the United Nations Convention on the Rights of Persons with Disabilities (UNCRPD), “persons with disabilities include those who have long-term physical, mental, intellectual or sensory impairments which in interaction with various barriers may hinder their full and effective participation in society on an equal basis with others” [3, p.4]. People with disabilities, particularly those who are living in LMICs, are one of the most vulnerable groups in society. They are at higher risk of poverty due to an interplay of higher medical expenditures, lower educational attainment, lower levels of health, and lower employment rates [4,5]. Additionally, people with disabilities in many LMICs often lack the necessary support both in the public and private sectors. For instance, the World Health Organization estimated that of the 75 million people who require a wheelchair only 5–15% have access due to persons with disabilities’ inability to afford the cost and absence of national assistive technology policies to cover the cost of assistive devices [6,7].

Given this, international policy frameworks, such as UNCRPD and the Convention on the Rights of the Child have recognized the role of government in supporting children with disabilities (CWDs) and their families' access to support in the public sector [3,8]. As mandated in the UNCRPD’s Article 25, people with disabilities have the right to equitable access to mainstream programs, including disability-specific programs such as rehabilitation services and assistive devices. Further, Article 28 of the UNCRPD highlights that people with disabilities have the right to equitable access to social protection programs such as financial assistance. Despite these, evidence suggests that people with disabilities experience barriers in accessing disability-specific programs offered by the government due to low coverage [9]. Evidence also indicates that disability assessment procedures sometimes create barriers for people with disabilities in accessing disability-specific programs. This is particularly a problem when eligibility criteria are not well defined or administrative capacity is insufficient to implement them properly [10].

There is increasing evidence that depending on the context, families of CWDs may encounter a wide range of barriers to accessing healthcare services. Although such contexts shape access barriers to healthcare services, commonalities exist which include shortage of providers and services, distance to care facilities, long wait times, limited transportation services, high costs of services, negative attitudes of providers, inaccessible built environments and limited knowledge about existing support [11–13]. For instance, Taderera and Hall (2017) [13] found that family members’ limited knowledge about disability-specific services (e.g., medical care, educational support, and daily living support) impeded their access to such services for their CWDs in Namibia. Evidence also suggests that family members’ and child’s sociodemographic characteristics such as family income, age, type of disability and location of their residence were associated with their access to healthcare services [9,14].

There is limited literature discussing family members' access to healthcare services for CWDs in Bangladesh, but the literature that does exist is in line with global evidence [11,15–18]. For instance, a recent epidemiological study that examined the prevalence of cerebral palsy in rural Bangladesh found that almost 80% (n = 568) of children with cerebral palsy never received rehabilitation services [17]. The authors further noted that the remaining 20% of children with cerebral palsy received rehabilitation services only from non-governmental organizations or hospitals [17]. The authors also reported that several factors deterred families in accessing rehabilitation services, including lack of knowledge, high cost of services, and inaccessible transportation [17]. An earlier quantitative study [15] found that families with higher socioeconomic status (e.g., income and literacy) and those who have children six years and older with physical impairment were more likely to take up referrals related to rehabilitation services for their CWDs. Additionally, evidence suggests that traditional beliefs about disability influence health-seeking behaviors of families of CWDs [18].

Previous research in Bangladesh has contributed to the knowledge on access to healthcare services and factors that affect service utilization among families of CWDs [11,15]; however, there are some limitations. For example, studies mostly focused on rehabilitation and treatment services offered in private hospitals and specialist non-governmental organization settings [15]. Moreover, studies focused on children with cerebral palsy and were also conducted in rural settings [16,17].

To our knowledge, no study has explored family members’ access to a wide range of disability-specific support offered by the government that is available free of cost for CWDs, and factors that may have influence their decisions to access such support in the public sector. Therefore, the current study explores family members’ knowledge about and access to different government supports (i.e., disability allowances, education, a stipend for education, rehabilitation services and reserved seats in public transportation) for CWDs in Bangladesh. The research questions are: (a) to what extent do families of CWDs have knowledge about government support?; (b) to what extent do families of CWDs have access to government support; and (c) what sociodemographic characteristics of families of CWDs and disability-related characteristics of CWDs are associated with access to government support?; and (d) what other factors (e.g., distance, cost of support) are associated with family members’ access to government support?

Understanding family members’ knowledge about and access to government support is critical to identify barriers that may deter them from accessing such support for their CWDs in a timely manner. Evidence suggests that timely access to appropriate support has the potential to prevent the development of complicated conditions that have a greater negative impact on CWDs, families, and society at large [19,20]. Additionally, evidence suggests that there is a need to generate more knowledge on access to disability-specific services for CWDs and their families in LMIC contexts because evidence from high-income countries is not directly contributing to improving the situation in LMICs [21]. As such, we believe that the context-specific findings of our study may be used to inform policy and provide guidance on how to best support these individuals.

### Bangladeshi context and government initiatives to provide support to CWDs

Bangladesh is one of the most densely populated countries in the world and a majority of its population lives in rural areas. Over the last few decades, Bangladesh has made remarkable progress in reducing poverty. Based on the international poverty line of $1.90 a day, it has made significant progress in reducing poverty from 44.2 percent in 1991 to 14.8 percent in 2016/17 [22]. Rapid growth has enabled Bangladesh to reach the lower middle-income country status in 2015. Despite this success, the country still faces daunting challenges, as approximately 24 million people are still living below the poverty line [22].

There is a lack of reliable information about the prevalence of disability in Bangladesh. Nevertheless, the estimated prevalence of disability in Bangladesh ranges from less than 1.4% [23] to 17.5% [24]. This difference in the two estimates may arise from the criteria used in defining disability. For example, persons with invisible disabilities (e.g., hearing impairment, developmental and learning disability, and those with mental health issues) are often excluded in the categorization of disability in many LMICs [25]. People with disabilities in Bangladesh are often deprived of their fundamental human rights in relation to health, education and other resources [24]. Furthermore, the stigma surrounding disability (e.g., disability is a curse, punishment of sin, and possession by ghosts or evil spirits) and discrimination at different levels of society appear to be major issues of violation of rights of persons with disabilities, including CWDs [18,26].

The government of Bangladesh has signed and ratified the UNCRPD and enacted the Rights and Protection of Persons with Disability Act in 2013 [27]. It has many provisions for supporting CWDs and their families. For instance, the Act affirms CWDs’ access to education, including the right to get reasonable accommodation. Further, it guarantees the rights of CWDs in accessing the same quality and standard of care (i.e., medical services, including rehabilitation) as provided to other persons. Moreover, the Act directs the inclusion of CWDs in the existing social safety net and poverty alleviation programs. It also mandates reserved seats for persons with disabilities, including children, in public transportation services and subsidized transportation fares.

In addition to policy commitments, the government has introduced disability-specific programs to meet the needs of CWDs and their families. For instance, the government has introduced a disability allowance—a monthly allowance of 700 taka (USD $8.3/month) that is given to CWDs. The government also offers rehabilitation services through the establishment of 103 rehabilitation service centers in all the 64 districts of Bangladesh. CWDs can receive physiotherapy, occupational therapy, and speech and language therapy services from those centers free of cost. Finally, the government has introduced a stipend for education for students with disabilities intending to ensure universal education for all [28]. Access to these government supports are based on eligibility criteria—in particular, CWDs must have a government-issued disability identification card [27]. CWDs under six years are not eligible for some of the government supports, including the disability allowance [29]. Similarly, CWDs are not qualified for the educational stipend unless they are enrolled in educational institutions recognized by the government [28]. However, there are no eligibility criteria required in terms of accessing rehabilitation services from the public sector.

#### Theoretical framework

To enhance our understanding of access and to guide us in preparing the survey questionnaire, we employed access to healthcare framework to examine family members’ access to government support in Bangladesh. The dimensions of the access framework we used are as follows: Awareness; Availability; Accommodation; Accessibility; Affordability; and Acceptability [30–33]. The framework guided development of the survey tool by providing us with factors that affect access to support. Specifically, the dimensions of access assisted us in identifying variables/indicators that were used to measure family members’ access to government support for CWDs. Variables such as knowledge, distance, wait time, and cost of support were added to the survey based on the framework. For example, we posed questions including, “*how far is the office of the government support center from your place of residence*?*” and “how long did you wait to get the government support from the time you applied for it*?*”* Table 1 provides an overview of each dimension.

**Table 1:** Dimensions of access.

Dimensions	Definitions
Availability	The volume and type of resources in relation to clients’ needs (e.g., providers, facilities, and services).
Accessibility	The location of services in relation to clients’ locations (e.g., transportation and geographical features like distance and climate).
Affordability	The costs of services and the clients’ ability to pay for both direct (e.g., health care) and indirect (e.g., transportation) costs associated in receiving the services.
Accommodation	The manner in which resources are organized in relation to the client’s needs (e.g., wait time, appointment systems, and hours of operation).
Acceptability	The characteristics and attitudes of providers and clients in relation to the characteristics of the services (e.g., social and cultural norms).
Awareness	The information and communication strategies among clients and providers about the health care system (e.g., health literacy).

## Materials and methods

### Study design and settings

We conducted a cross-sectional survey [34] among family members of CWDs in Bangladesh who sought rehabilitation services for their CWDs from the Centre for the Rehabilitation of the Paralysed (CRP) between December 2018 to February 2019. CRP is a non-profit organization that has offered a wide range of services to persons with disabilities, including children (e.g., children with cerebral palsy, autism spectrum disorder, speech problems) and their families since 1979. We recruited participants primarily from two divisional centers of CRP (i.e., Savar and Rajshahi). Families of CWDs who were living in the northern part of Bangladesh and were not able to travel to the Savar Center can seek services at CRP-Rajshahi. Although similar services are available at both centers, services at CRP-Savar are more comprehensive compared to CRP-Rajshahi. For instance, CRP-Savar offers a two-week residential program as well as appointment-based services to CWDs and their families. Depending on the child’s condition, either a child is admitted for a two-week residential rehabilitation program or an appointment is made at the outpatient unit. Further, professionals working at CRP-Savar organize comprehensive parental education on disability once a week on a regular basis. Parental education mostly covers information on disability, home management strategies and support that are available for CWDs both in public and private sectors. The two-week residential program and the educational program for families are not available at CRP-Rajshahi [35].

### Study participants and sampling

The study participants were family members of CWDs. We defined family member any person(s) “who regard themselves as a family and who carry out the functions that families typically perform. These people may or may not be related by blood or marriage and may or may not usually live together” [36]. This definition is appropriate within Bangladeshi context in that CWDs are sometimes raised by extended families or adopted families. A sample size of 393 was calculated based on estimated prevalence equation which is n = [(z^2^)*P*(1-*P*)]/d^2^ considering the proportion of 50% (maximum uncertainty principle) [37]. We employed a convenience sampling technique to recruit participants from CRP-Savar and CRP-Rajshahi. Inclusion criteria were as follows: (a) being a family member of a CWD; (b) above 18 years of age; (c) willingness to participate; and (d) ability to communicate in Bengali.

### Survey tool/questionnaire development and validation

We developed a structured questionnaire encompassing demographic characteristics of participants and their accompanied CWDs, participants’ knowledge about government support, and access to such support. The survey questionnaire was adapted from Devkota and colleagues [38] and was also guided by the access framework indicated earlier. Table 2 provides an example of sample questions.

**Table 2:** Example of survey questions.

1. Please choose the government support available for your child with a disability that you are knowledgeable about (check all that apply to you).				
a. Disability allowance	◻ Yes	◻ No	◻ Uncertain	
b. Inclusion in the mainstream school	◻ Yes	◻ No	◻ Uncertain	
c. Education stipend	◻ Yes	◻ No	◻ Uncertain	
d. Free rehabilitation services	◻ Yes	◻ No	◻ Uncertain	
e. Reserved seats in public transports	◻ Yes	◻ No	◻ Uncertain	
2. Please choose the most appropriate description regarding the actual use of government support for your child with a disability.				
a. Disability allowance	◻Currently using it	◻ Used it in the past	◻ Tried but failed to get it	◻Never tried to get it
b. Inclusion in the mainstream school	◻Currently using it	◻ Used it in the past	◻ Tried but failed to get it	◻Never tried to get it
c. Education stipend	◻Currently using it	◻ Used it in the past	◻ Tried but failed to get it	◻Never tried to get it
d. Free rehabilitation services	◻Currently using it	◻ Used it in the past	◻ Tried but failed to get it	◻Never tried to get it
e. Reserved seats in public transports	◻Currently using it	◻ Used it in the past	◻ Tried but failed to get it	◻Never tried to get it
3. How far (in kilometers) is the office of the government support from your place of residence?				
4. How long (in minutes) does it take to get to the office of government support?				
5. How long (in days) did you wait to get the government support from the time you applied for it?				
6. Did you pay any direct cost to get the support? If yes, please specify the amount.				
7. Did you pay any indirect cost to get the support? If yes, please specify the amount.				
8. Is the government facility accessible for persons with mobility devices?				
9. What is your level of satisfaction with the attitude of the providers?				

We developed the survey questionnaire in English and checked it with two independent researchers, who are experts in measuring access to health care for vulnerable population, to ensure its face validity [39]. The questionnaire was then translated from English to Bengali and then back translated into English by three translators, including the first author, who is fluent in both English and Bengali, to ensure consistency between the Bengali and English version. The Bengali questionnaire was then pilot-tested with 10 participants using a set of guided questions such as “Are the instructions clear enough? Are questions easy to understand?” to ensure that the content was understandable.

### Data collection

We collected data with the help of trained research assistants (n = 4) via one-on-one in-person structured interviews and the first author monitored the data collection process. In person interviews with families was appropriate in the Bangladeshi context given that the postal service is inefficient in this context. This method of data collection enabled us to maximize response rate and to minimize missing value [40]. The research assistants approached family members while they were waiting for their rehabilitation service at CRP, they explained the purpose of the survey to the participants and asked for their voluntary participation. For those who agreed to participate, the research assistants surveyed them in-person at a mutually agreed time and place. It took approximately 20 minutes for the research assistants to complete a survey with each participant. The first author checked the surveys for completeness, accuracy, clarity, and consistency.

### Data management

#### Access to government support

“Government support” represents (a) disability allowance, (b) inclusion in mainstream school, (c) educational stipend, (d) rehabilitation services implemented under the Ministry of Social Welfare, and (e) reserved seats in public transportation. This list of government support was developed from those described in the Rights and Protection of Persons with Disability Act of 2013 and were identified by families in an earlier study in the Bangladeshi context [41]. Access to each government support was assessed using four categories: *currently using it*, *used it in past*, *tried but failed to get it*, *and never tried to get it* (see the sample questions in Table 2). When a participant responded to any of the following options: *currently using it*, *used it in past or tried but failed to get it* a follow up question was posed for other indicators across various dimensions of access: availability, affordability, accessibility, accommodation and acceptability. For the respondents who indicated that they *“never tried”* to access the support, the follow up questions were skipped.

For statistical analysis, participants’ access to each support, listed earlier, was counted as “yes” if a response of either “used it in the past” or “currently using it” was given. A response of “no” access was counted if a response of “tried but failed to get it” or “never tried to get it” was given. As such, we had five binary (yes/no) items in relation to participants’ access to government support. When participants' responses were “yes” for at least one out of the five government support, we further categorized them as have “access to at least one support” and the remaining participants were grouped as having “no access.”

#### Knowledge about government support

We assessed family members’ knowledge about each support through subjective indicators using three categories: *yes*, *no* and *uncertain* to the question “please choose the government support available for your child with a disability that you are knowledgeable about”. However, we recoded these three categories as a binary variable using *yes* and *no* (including *no* and *uncertain*). We collapsed “*uncertain*” under “*no”* category because of small sample size in “*uncertain*” stratum. As such, we had five binary (yes/no) items in relation to participants’ knowledge about five different government support indicated earlier. If participants knew about at least three out of five support, we further grouped them as having “*adequate knowledge”* and the reaming participants were grouped as having “*limited knowledge*.*”*

#### Disability of the child

The nature of the child’s disability was identified through self-report from the family member and was verified through two sources: the participant’s disability identification card issued by the government of Bangladesh (where available), and the therapy appointment card issued by a team of rehabilitation professionals at CRP. For statistical analysis, the child’s disability was recoded into three categories: Cerebral palsy, Autism Spectrum Disorder, and Other. The “Other” forms of disability consisted of those who were diagnosed with Down syndrome, speech disability, physical disability, spina bifida, and multiple disabilities. We collapsed them together because of the small number in each category.

### Statistical analysis

We performed both descriptive and inferential statistical analyses to examine the interaction among the variables affecting access and p-value of 0.05 as the cut-off for statistical significance using SPSS version 17.0 for analysis. We performed descriptive statistics (e.g., percentage, minimum and maximum value) to summarize different continuous as well as categorical variables. We used chi-square (χ^2^) test for comparisons between categorical variables within different subgroups of the study participants [42]. Specifically, we computed chi-square (χ^2^) test for independence to determine the significance of differences in participants’ knowledge about and access to different government support across different subgroups of participants. We divided participants into subgroups based on age, sex, education, monthly family income, residence area, types of disability of accompanied CWDs and age of CWDs. For continuous variables that did not have normal distribution, we used Mann Whitney U test to compare mean ranks among sub-groups.

### Ethical consideration

We obtained ethical approval from the Queen’s University Health Sciences and Affiliated Teaching Hospitals Research Ethics Board (HSREB) in Canada (Reference number: 6024484) and the Ethical Review Board of CRP (study site) in Bangladesh (Reference number: CRP-R&E-0401-231).

## Results

### Response rate and number of participants

We invited a total of 399 family members of CWDs to take part in this survey and 393 participated. The response rate was 98.6%. Of the 393 participants, 73% sought services from CRP-Savar and 27% from CRP-Rajshahi. Although it was not expected, these two groups were significantly different in terms of their demographic and socioeconomic characteristics. Compared with CRP-Rajshahi, participants from CRP-Savar were more likely to be younger (< 30 years) (51.6% vs 37.7%, p <0.01), more females (85.7% vs 74.5%, p < 0.001), more from rural area (58.2% vs 33.0%, p < 0.001), less likely to have higher education (17.1% vs 43.4%, p < 0.001) and less likely to be in high-income category (9.8% vs 32.1%, p < 0.001).

### Demographic characteristics of the participants

Table 3 summarises demographic characteristics of participants and their CWDs. A majority of the participants were below 30 years, female, and the biological mother of the CWD. More than half of the participants were educated to secondary or college level. The place of residence distribution was almost equal in rural and urban areas. More than one third of participants were from the low-income category. In terms of the CWD’s characteristics, over half were reported to be less than six years old and were boys. Two-thirds of the CWDs were diagnosed with Cerebral Palsy, almost a quarter of CWDs were categorized as having a severe disability, and three-quarters of the CWDs did not use any mobility device at the time of the survey.

**Table 3:** Demographic characteristics of participants (n = 393).

Family characteristics	n (%)	Child characteristic	n (%)
**Age groups**		**Age groups**	
< 30 years	188 (47.8)	< 6years	213 (54.2)
30 to 40 year	152 (38.7)	≥ 6 years	180 (45.8)
> 40 years	53 (13.5)	**Sex**	
**Sex**		Girl	151 (38.4)
Female	325 (82.7)	Boy	242 (61.6)
Male	68 (17.3)	**Types of disability**	
**Educational level**		Cerebral Palsy	268 (68.2)
No or primary education	65 (16.5)	Autism Spectrum Disability	80 (20.4)
Secondary or college education	233 (59.3)	Other	45 (11.5)
Graduate or equivalent	95 (24.2)	**Mobility devices**	
**Residence**		None	300 (76.3)
Rural	202 (51.4)	Special seat	23 (5.9)
Urban	191 (48.6)	Wheelchair	21(5.3)
**Occupation**		Walking frame	15 (3.8)
Housewife	290 (73.8)	Ankle foot orthosis	34 (8.7)
Formal employment	42 (10.7)	**Severity of disability**	
Business	31 (7.9)	Mild	40 (10.2)
Daily laborer/Farmer	12 (3.1)	Moderate	260 (66.2)
Retired person/senior	12 (3.1)	Severe	93 (23.7)
Unemployed	6 (1.5)	**Monthly family income**	
**Relation to the child**		Low (≤10,000 taka)	136 (34.6)
Mother	293 (74.6)	Medium (10,001–30,000 taka)	195 (49.6)
Father	65 (16.5)	High (>30,000 taka)	62 (15.8)
Brother/Sister	2 (0.5)		
Aunt/Uncle	10 (2.5)		
Grandparent	23 (5.)		

### Knowledge about and access to government support and their association with demographic characteristics

#### Knowledge about government support

Of the 393 participants, 56.2% had adequate knowledge about government supports. We found a significant association between participants’ knowledge about government support and the location of CRP services they had access to (χ^2^ = 90.87, p < 0.001). Compared to CRP-Rajshahi, participants from CRP-Savar were more likely to have adequate knowledge about government support (70.7% vs 17.0%). We also found a significant association between participants’ knowledge and age (χ^2^ = 13.164, p < 0.001) and type of disability of their accompanied CWDs (χ^2^ = 4.07, p = 0.04). Participants with CWDs aged six and above were more likely to have adequate knowledge about government support compared to those with child aged less than six years (66.1% vs 47.9%). Similarly, participants with CWDs diagnosed with Cerebral Palsy were more likely to have adequate knowledge about government support compared to their counterparts of CWD diagnosed with Autism Spectrum Disability (59.0% vs 46.3%).

However, participants’ knowledge was not significantly associated with other demographic characteristics of the participants (e.g., sex, level of education, area of residence, and monthly family income), and type and severity of disability of the child (see Table 4).

**Table 4:** Demographic characteristics and their association with knowledge about and access to government support (n = 393).

	Knowledge about government support		χ^2^	p-value	Access to government support		χ^2^	p-value
Demographic characteristics	Adequate knowledge [n (%)]	Limited knowledge [n (%)]			Accessed at least one support [n (%)]	No access [n (%)]		
**Participants’ demographic characteristics**								
**Location of CRP services**								
Savar	203 (70.7)	84 (29.3)	90.87	<0.001	125 (43.6)	162 (56.4)	0.75	0.38
Rajshahi	18 (17.0)	88 (83.0)			41 (38.7)	65 (61.3)		
**Sex**								
Female	181 (55.7)	144 (44.3)	0.22	0.63	134 (41.2)	191 (58.8)	0.78	0.37
Male	40 (58.8)	28 (41.2)			32 (47.1)	36 (52.9)		
**Education level**								
No or primary education	37 (56.9)	28 (43.1)	0.11	0.94	33 (50.8)	32 (49.2)	2.46	0.29
Secondary or college education	132 (56.7)	101 (43.3)			96 (41.2)	137 (58.8)		
Graduate or equivalent	52 (54.7)	43 (45.3)			37 (38.9)	58 (61.1)		
**Residence**								
Rural	115 (56.9)	87 (43.1)	0.08	0.77	87 (43.1)	115 (56.9)	0.11	0.73
Urban	106 (55.5)	85 (44.5)			79 (41.4)	112 (58.6)		
**Monthly family income**								
Low (≤10,000 taka)	74 (54.4)	62 (45.6)	5.09	0.07	59 (43.4)	77 (56.6)	0.80	0.67
Medium (10,001–30,000 taka)	119 (61.0)	76 (39.0)			84 (43.1)	111 (56.9)		
High (>30,000 taka)	28 (45.2)	34 (54.8)			23 (37.1)	39 (62.9)		
**Demographic characteristics of CWDs**								
**Sex**								
Girl	84 (55.6)	67 (44.4)	0.03	0.84	58 (38.4)	93 (61.6)	1.47	0.22
Boy	137 (56.6)	105 (43.4)			108 (44.6)	134 (55.4)		
**Age of the child**								
<6years	102 (47.9)	111 (52.1)	13.16	<0.001	71 (33.3)	142 (66.7)	15.11	<0.001
≥6 years	119 (66.1)	61 (33.9)			95 (52.8)	85 (47.2)		
**Type of disability of CWDs**								
Cerebral palsy	158 (59.0)	110 (41.0)	4.037	0.04	116 (43.3)	152 (56.7)	2.31	0.12
Autism Spectrum Disorder	37 (46.3)	43 (53.8)			27 (33.8)	53 (66.3)		
Other*	26 (57.8)	19 (42.2)			23 (51.1)	22 (48.9)		
**Severity of disability**								
Mild	21 (52.5)	19 (47.5)	1.354	0.50	15 (37.5)	25 (62.5)	3.16	0.20
Moderate	143 (55.0)	117 (45.0)			118 (45.4)	142 (54.6)		
Severe	57 (61.3)	36 (38.7)			33 (35.5)	60 (64.5)		

*was excluded from statistical analysis

#### Access to government support

Of the 393 participants, only 42.2% had access to government support. Participants’ access to government support was not significantly associated with their demographic characteristics and that of their accompanied CWDs except for the age of the child (χ^2^ = 15.118, p < 0.001). Participants with CWDs aged six and above were more likely to have access to government support compared to those with CWDs aged less than six years (52.8 vs 33.3%) (see Table 4).

#### Access to government support and various dimensions of access framework

We found a significant association between knowledge about and access to government support (p < 0.001). Compared to the participants who had limited knowledge about government support, participants who had adequate knowledge were more likely to have access to such support (30.1% vs 69.9%). We also found a significant association between accessibility of public premises and access to government support (p < 0.01). Although a majority of participants (72.9%) reported that the public premises were not accessible, participants who reported an accessible environment were more likely to have access to government support compared to those who reported an inaccessible environment (69.2% vs 53.3%). There was also a significant difference in terms of wait time between the two groups: those who accessed at least one government support and those who had no access. Participants who did not have access to government support had to face longer wait times compared to their counterparts. We did not find any significant difference in terms of distance and time to reach the support center and cost of support between these two groups (see Table 5).

**Table 5:** Access inequalities across access dimensions.

χ^2^ test for categorical variables	Access to government support		χ^2^	p-value
	Accessed at least one support (n (%))	No access (n (%))		
Knowledge (n = 393)				
Adequate knowledge	116 (69.9)	105 (46.3)	21.74	<0.001
Poor knowledge	50 (30.1)	122 (53.7))		
Accessibility of public premises (n = 288)				
Yes	54 (32.5)	24 (19.7)	5.88	0.01
No	112 (67.5)	98 (80.3)		
Clients satisfaction with providers’ (n = 282)				
Satisfied	102 (61.8)	66 (56.4)	0.83	0.36
Dissatisfied	63 (38.2)	51 (43.6)		
**Mann Whitney u test (z) for continuous variables**	**Accessed at least one support (Mean rank)**	**No access (Mean rank)**	**Mann Whitney U test**	**p-value**
Wait time (day) to get support (n = 237)	110.8	132.08	5453.0	0.02
Distance to support center (kilometer) (n = 289)	152.2	135.18	9001.0	0.08
Time to reach the location of support (n = 289)	148.8	139.78	9566.5	0.35
Direct cost (n = 65)	31.9	36.5	322.5	0.41
Indirect cost (n = 208)	96.36	86.86	3268.0	0.27

NB: n is different across dimensions because all dimensions were not applicable to all participants. Questions across access dimensions were skipped for participants who never tried to get support.

## Discussion

The purpose of this study was to examine the extent to which families of CWDs have knowledge about and access to different government supports in Bangladesh. We employed access dimensions as the theoretical lens to understand the phenomena, which is exploring the factors that affect access to government support. We specifically found that participants’ knowledge (Awareness), wait time (Accommodation), and navigating the built environment (Accessibility) were associated with access to government support. However, we did not find any association between cost of services (Affordability) and family members’ access to government support. Specifically, the analyses uncovered the following major findings, which are discussed in the following paragraphs: (a) families with CWDs have limited knowledge about and access to government support; (b) family members’ knowledge about government support was associated with the location of the CRP centre where they sought services for their CWDs; and (c) family members’ access to government support was associated with their knowledge about government support, and age of their CWDs.

### Knowledge inequalities

Similar to previous studies that were conducted in rural Australia [43] and other LMICs contexts [13,44], families of CWDs in our study reported limited knowledge about government support. Almost half of the participants were not aware of government supports that were available for CWDs. Participants who had access to CRP services at Savar were more likely to have adequate knowledge about government support although these participants were less likely to have higher education compared to participants from CRP-Rajshahi. One possible explanation could be the comprehensive nature of service that is offered to CWDs and their families in Savar. In particular, professionals in Savar not only offer services to CWDs but also make efforts to raise awareness among families about disability. Specifically, rehabilitation professionals invite both parents in a weekly meeting and discuss topics about disability and government support that are available for CWDs in Bangladesh. Such awareness programs can be valuable for families and have the potential to facilitate access to a wide range of supports for CWDs [18]. Thus, the findings highlight the potential value of ongoing awareness programs for families who do not have access to CRP services. It may be advisable that similar family education programs be offered at all CRP centers, rather than just at the headquarter. It should not just be the responsibility of non-governmental organizations to increase families’ awareness of national supports. Rather, the government should strengthen efforts towards increasing awareness among families, service providers (e.g., health and education) and others who are working with CWDs. There are a wide variety of formats of disability awareness interventions for families of CWDs including written materials (e.g., pamphlet), videos, dramas, theatres and puppet shows, and discussion [45]. Such interventions are critical to enhance knowledge about disability and facilitate access for CWDs to their needed support, including health [46,47]. For instance, a study in Bangladesh found that school enrolment rates for CWDs increased in areas where awareness programs for parents and teachers were available compared to the areas with no such interventions [47]. Further, increasing awareness about disability and government support amongst service providers (e.g., health and education) and community workers is critical in that these service providers often serve as the first point of contact for families of CWDs when families are seeking assistance. The government should organize in-service training or sensitization meetings on a regular basis for service providers so that they can better inform and assist their clients to navigate public system. It would also be interesting to integrate disability awareness in the health training institutions' academic curricula.

### Access inequalities

Similar to knowledge, families of CWDs had limited access to government support for their CWDs. More than fifty percent of the participants did not have access to government support at all. The findings of this study are congruent with previous research in other countries indicating that limited availability of services in LMICs impeded CWDs’ access to services in the public sector [14,48]. For instance, Fisher and Shang (2013) [14] found that CWDs have limited access to government support in China, and therefore they mostly relied on their families for support and care. Limited access to appropriate support, including health service, can have a negative impact on CWDs as well as other family members. For example, limited access to health services among persons with disabilities, including children, may increase the risk of physical and mental health issues (e.g., challenging behavior), and also decrease social participation [19]. Further, evidence suggests that a lack of access to appropriate services can place CWDs at a greater risk of poverty [20]. To avoid these consequences, there is a need for policy intervention to maximize support for CWDs in the public sector, and also tackle barriers that impede family members in accessing such support. The government also need to allocate more resources for additional support and/or personnel to meet various needs (e.g., health, education and finance) of families of CWDs. Future studies need to explore family members’ experiences with a view to identify barriers and facilitators in accessing government support particularly in Bangladesh. It would also be interesting to explore the impact of receiving different government supports.

Although it is not surprising, we found that participants who had adequate knowledge about government support were more likely to access support. This finding is consistent with previous studies where authors found a positive relationship between awareness and the use of services indicating high awareness is associated with high use of services [49,50]. Recent evidence also shows that families of CWDs who had limited knowledge encountered more challenges in accessing services [13]. Thus, the finding underscores the need for ongoing awareness programs among families of CWDs as indicated earlier.

In our survey, family members of older children tended to have better access to government support than those with younger children. Conversely, a previous study found that younger children tend to have better access to rehabilitation services in Canada [51]. The possible reason may be due to the fact that children less than six years are not eligible for some government support in Bangladesh, in particular the disability allowance [29]. The benefits of early intervention have been extensively discussed in the literature. It is evident that not only CWDs and their families but also the state will greatly benefit when interventions are initiated for younger children [52,53]. For instance, Koegel (2000) [52] found that children who were completely non-verbal and began an intervention in the early pre-school years were more likely to become verbal than children who began intervention over the age of 5-years. Further, evidence suggests that early intervention leads to fiscal savings by reducing the negative effects of a disabling condition over time that often require costly interventions [54]. Given this, the government of Bangladesh should make it a priority for families to have greater access earlier on and this will, in turn, save taxpayers’ money in the long term. Thus, the finding underscores the need for policymakers to revise that policy clause and facilitate CWDs in accessing their needed support, including disability allowance, as early as possible. Evidence suggests that access to disability allowance can facilitate access to other services such as health and education for CWDs [55]. In view of this, service providers (e.g., doctors, nurses, rehabilitation professionals and community workers) need to help families to learn how to seek assistance and find appropriate services to suit their child in the early stage.

Although families from the low-income category should, in theory, have priority in accessing government support according to the Rights and Protection of Persons with Disability Act of 2013 [27], we did not find any significant differences among different economic groups in terms of the actual access to government support. Our results highlighted a general gap in the translation of disability policy to practice. Findings of our study indicate the need to reform the current government support system to prioritize access to support for families with the greatest needs. However, it is challenging to reach the most marginalized groups particularly in a context of low coverage of social protection schemes like Bangladesh. Low coverage increases competition among applicants and thus holds greater risk for inequitable allocation of coverage based on nepotism, bribery, patronage and political gain [10,56]. Thus, there is a need for strong monitoring and supervision systems, and also strong political commitment to achieve the goal of each social protection scheme [10]. Further, evidence suggests that the involvement of Disabled Peoples’ Organizations in the selection process and also strengthen the capabilities of disabled people to advocate for their entitlements could potentially ensure robust and fair selection processes for government support [57]. Additionally, evidence suggests that higher coverage of social protection schemes and investment in creating public awareness could better reach the individuals with the greatest needs [10].

However, our findings in relation to income and access to support is different from a previous study in Bangladesh where the authors found that families of CWDs with higher socioeconomic status were more likely to take up referrals for disability-specific services for their CWDs [15]. The positive relationship between income and access to services was also found in previous research that were conducted in high-income settings [58,59]. For instance, Beatty and colleagues (2003) [58] found that persons with disabilities with lower incomes are less likely to receive health services. The differences in findings could be due to the limitation in our sample selection as described below.

We also observed a significant difference in terms of wait time between family members who had access and who did not have access to government support. In particular, we found that families who did not have access reported longer wait times to receive supports. This finding also concurs with another study that was conducted in rural Australia, indicating a long wait time to get access to government support for CWDs [60]. Evidence suggests that long wait times to access needed support can have a negative impact on the psychosocial quality of life of CWDs [61]. Thus, there is a need for policymakers to identify strategies and solutions for reducing wait times. However, long wait times to get disability-specific services is an issue around the world [51,60]. For instance, in a prospective cohort study by Feldman and colleagues (2002) [51] the average waiting time to get occupational and physical therapy in Canada was around four to five months. Evidence from high and low-income countries suggests that telehealth services, group activities and community interventions may reduce long wait times for therapy services [43,62]. Our analysis also supports these strategies; a recent study in Bangladesh found that the provision of online parent training programs is effective in enhancing parental knowledge about disability, and to provide direct support to CWDs in particular those with autism and neurodevelopmental disabilities [63].

Finally, reflecting on the conceptualization of access, the access framework was helpful in identifying factors that affected family members’ ability to access government support. In particular, framing the survey tool across the dimensions of access framework revealed specific information that we could have otherwise overlooked. Thus, this strategy helped us to answer our research questions. As such, researcher may consider using the access framework in understanding access to government support for CWDs in other contexts.

### Actionable recommendations

The findings of this study indicate that families of CWDs have limited knowledge about and access to government support. Thus, there is a need for the government to develop strategies to communicate information to families of CWDs. In particular, evidence suggests the need for context-specific adaptation of communication strategies to reach people who are illiterate or have a type of impairment [10]. The government should invest in conducting vigorous awareness campaigns through mass media such as television and radio to enhance families’ level of awareness of government support. Furthermore, it would be most appropriate if community health workers can visit families of CWDs and make them aware of available government support [64]. Alternatively, announcements can be made in places of worship (i.e., mosques, churches, and temples) as they are recognized community gathering places particularly in rural areas [65]. Evidence also suggests that the involvement of Disabled People Organizations in disseminating information about disability targeted programs will increase the reach of this message to potential beneficiaries with a disability and their families [44]. Moreover, there is a need for the government of Bangladesh to invest more resources to increase coverage of government support for CWDs. In particular, the government may follow strategies such as multisectoral collaboration (e.g., Ministries of Social Welfare, Ministry of Health and Ministry of Education) to raise funds for disability-specific programs [66]. Finally, it is critical to prioritize CWDs under six years of age for all forms of government support, especially the disability allowance. This will, in turn, help families to offset the cost associated with accessing services for CWDs.

## Limitations

Although this study involved a large sample of family members of CWDs, the results and implications should be considered in light of several methodological limitations. First, this was a cross-sectional study and as such the direction of effects (e.g., knowledge versus access) cannot be determined. Second, participants were drawn from a convenience sample from a rehabilitation center and therefore the sample may not be representative of all families with CWDs of Bangladesh. This limitation challenges the generalisability of the findings. As such, we suggest that future studies can incorporate consultation with families who are living in the community and do not have access to CRP services. In particular, a community-based survey is needed to reach underserved populations. Finally, this study solely relied on participants’ self-reported measures (e.g., family income and waiting time to get government support) and, therefore, may be influenced by recall and reporting bias. To minimize recall bias, we used strategies, such as pilot-testing the questionnaire to make sure the questions could be easily understood. Further, we provided training and daily supervision to the research assistants to ensure quality in data collection.

## Conclusion

Despite these limitations, this study has important implications that should be considered. The results point to Bangladeshi family members’ lack of knowledge about and limited access to government supports for their CWDs. A key point of this study is that participants’ awareness of and access to government support for CWDs needs to be increased. By providing access to government support, families of CWDs will be better able to meet the complex needs of their CWDs. The development of parental education similar to that which is provided at CRP-Savar could help to increase knowledge of disability amongst families of CWDs living in the community and do not have access to CRP services. The results of the study have other implications for policy, practice and research. We believe that these findings will guide policymakers to initiate policy interventions toward improving knowledge about and access to government support for CWDs in Bangladesh. We also highlighted the need for in-service training for service providers about disability so that they can disseminate information among families about government support. They can also facilitate families’ access to such support by referring them to the appropriate locations. It will be important for future studies to uncover more in-depth depictions of family members’ experiences in accessing government support to provide deeper understanding of the disability-related support access barriers and facilitators experienced by families in Bangladesh.

## Supporting information

S1 Appendix Survey questionnaire. (DOCX)
