# Supplementary material for: Exploring access to government-led support for children with disabilities in Bangladesh
Source: PLoS One. 2020 Jul 2;15(7):e0235439. doi: 10.1371/journal.pone.0235439 (PMC7332059; doi:10.1371/journal.pone.0235439)
Supplement: S1 Appendix — (DOCX) [file pone.0235439.s001.docx]

**Survey Questionnaire**

Serial Number: ……………….

Participant ID: ……………….. Interviewer’s Initial: …………………

Location of interview: CRP-Savar CRP-Rajshahi

**Part 1: Demographic characteristics of family members**

| Variables | Categories |
| --- | --- |
| Age | …………… years old |
| Sex | 🗌 Male 🗌 Female |
| Living area | 🗌 Rural 🗌 Urban |
| District | ------------------- |
| Education | 🗌 No formal education 🗌 Primary (grade I-V)  🗌 Secondary (grade VI-X) 🗌 Higher Secondary (grade XI-XII)  🗌 Bachelor/Master/PhD |
| Occupation | 🗌 Housewife 🗌 Daily laborer  🗌 Service holder 🗌 Unemployed  🗌 Retired person 🗌 Other (specify) ----------------- |
| Number of family members living with the child | ------------------- |
| Monthly family income | -------------------/taka |
| Relationship to the child with a disability | 🗌 Mother 🗌 Father 🗌 Brother/Sister  🗌 Aunt/Uncle 🗌Grandparent 🗌Other (specify) ------------------ |

**Part 2: Demographic characteristics of the child with a disability**

| **Variables** | **Categories** |
| --- | --- |
| Age | …………… months …………..years old |
| Sex | 🗌 Boy 🗌 Girl |
| Education | 🗌 Primary (grade I-V) 🗌 Secondary (grade VI-X)  🗌 Higher Secondary (grade XI-XII) 🗌 Never been to a school  🗌 Not applicable (child is not at school going age) |
| Type of disability | 🗌 Cerebral palsy 🗌 Autism/intellectual disability 🗌 Down syndrome 🗌 Speech disability  🗌 Physical disability 🗌 Mental illness leading to disability  🗌 Visual disability 🗌 Hearing disability 🗌 Deaf-blindness 🗌Multiple disabilities 🗌 Other disability |

**Part 3: Questions specific to family members’ knowledge about and utilization of the government support services.**

3.1 From the list below, please choose the government support available for your child with a disability that you are knowledgeable about (check all that apply to you).

| Government support services | Answer options | | |
| --- | --- | --- | --- |
| 1. Disability allowance | 🗌 Yes | 🗌 No | 🗌 Uncertain |
| 1. Inclusion in the mainstream schools | 🗌 Yes | 🗌 No | 🗌 Uncertain |
| 1. Education stipend | 🗌 Yes | 🗌 No | 🗌 Uncertain |
| 1. Rehabilitation services | 🗌 Yes | 🗌 No | 🗌 Uncertain |
| 1. Reserved seats in public transportations | 🗌 Yes | 🗌 No | 🗌 Uncertain |

3.2 From the list below, please choose the most appropriate description regarding the actual use of government support for your child (check all that apply to you)?

| Government support services | Answer options | | | |
| --- | --- | --- | --- | --- |
| 1. Disability allowance | 🗌 Currently using it | 🗌 Used it in the past | 🗌 Tried but failed to get it | 🗌 Never tried to get it |
| 1. Inclusion in the mainstream school | 🗌 Currently using it | 🗌 Used it in the past | 🗌 Tried but failed to get it | 🗌 Never tried to get it |
| 1. Education stipend | 🗌 Currently using it | 🗌 Used it in the past | 🗌 Tried but failed to get it | 🗌 Never tried to get it |
| 1. Rehabilitation services | 🗌 Currently using it | 🗌 Used it in the past | 🗌 Tried but failed to get it | 🗌 Never tried to get it |
| 1. Reserved seats in public transports | 🗌 Currently using it | 🗌 Used it in the past | 🗌 Tried but failed to get it | 🗌 Never tried to get it |

**Part 4: Questions specific to access dimensions**

In the section below, I will ask you some questions in accessing the government support, you checked earlier, across the dimensions of access framework. You can skip any question that is not applicable for you.

4.1 Disability allowance

| Questions | Answer options | | | |
| --- | --- | --- | --- | --- |
| 1. How far (in kilometers) is the office that disburses disability allowance from your place of residence? | …………………… kilometers | | |  |
| 1. How long (in minutes) does it take to get to the office that disburses disability allowance? | ………………….. minutes | | |  |
| 1. How long did you wait to receive the disability allowance from the time you applied for it? | ………………….. days | | |  |
| 1. Do/did you pay any direct cost to get the allowance? | 🗌 Yes |  | 🗌 No | 🗌Uncertain |
| 1. If yes, please specify the amount. | ................................. /taka | | | |
| 1. Do/did you pay any indirect cost in getting the allowance (e.g., cost of transportation)? | 🗌 Yes |  | 🗌 No | 🗌 Uncertain |
| 1. If yes, please specify the amount | .................................taka | | | |
| 1. Is the facility accessible for persons with mobility devices? | 🗌 Yes 🗌 No 🗌 Uncertain | | | |
| 1. What is your level of satisfaction with the provider’s attitude that disburses allowance? | 🗌 Extremely dissatisfied 🗌 Dissatisfied  🗌 Neutral 🗌 Satisfied  🗌 Extremely satisfied | | | |

4.2 Inclusion in mainstream school

| Questions | Answer options | | | |
| --- | --- | --- | --- | --- |
| 1. How far (in kilometers) is the school from your place of residence? | …………………… kilometers | | |  |
| 1. How long (in minutes) does it take to get to the school? | ………………….. minutes | | |  |
| 1. How long between when you initially applied for acceptance at school did your child receive permission to attend? ? | ………………….. days | | |  |
| 1. Do/did you pay to admit your child in the school? | 🗌 Yes |  | 🗌 No | 🗌 Uncertain |
| 1. If yes, please specify the amount | .................................. /taka | | | |
| 1. Do/did you pay any indirect cost to get your child to the school (e.g., cost of transportation)? | 🗌 Yes |  | 🗌 No | 🗌 Uncertain |
| 1. If yes, specify amount (per month) | .............................. taka | | | |
| 1. Is the school premises accessible for persons with mobility devices? | 🗌 Yes 🗌 No 🗌 Uncertain | | | |
| 1. What is your level of satisfaction with teachers’ attitude? | 🗌 Extremely dissatisfied 🗌 Dissatisfied  🗌 Neutral 🗌 Satisfied  🗌 Extremely satisfied | | | |

4.3 Stipend for education

| Questions | Answer options | | | |
| --- | --- | --- | --- | --- |
| 1. How far (in kilometers) is the office that disburses stipend from your place of residence? | …………………… kilometers | | |  |
| 1. How long (in minutes) does it take to get to the office that disburses stipend? | ………………….. minutes | | |  |
| 1. How long (in day) did you wait to enlist your child for the stipend from the time you applied for it? | ………………….. day(s) | | |  |
| 1. Do/did you pay any direct cost to get the stipend? | 🗌 Yes |  | 🗌 No | 🗌 Uncertain |
| 1. If yes, specify amount | ..................................taka | | | |
| 1. Do/did you pay any indirect cost in getting the stipend (e.g., cost of transportation)? | 🗌 Yes |  | 🗌 No | 🗌 Uncertain |
| 1. If yes, specify amount (per month). | ................................ /taka | | | |
| 1. Is the building accessible for persons with mobility devices? | 🗌 Yes 🗌 No 🗌 Uncertain | | | |
| 1. What is your level of satisfaction with person’s attitude who disburses the education stipend ? | 🗌 Extremely dissatisfied 🗌 Dissatisfied  🗌 Neutral 🗌 Satisfied  🗌 Extremely satisfied | | | |

4.4 Rehabilitation services

| Questions | Answer options | | | |
| --- | --- | --- | --- | --- |
| 1. How far (in kilometers) is the rehabilitation center from your place of residence? | …………………… kilometers | | |  |
| 1. How long (in minutes) does it take to get to the rehabilitation center? | ………………….. minutes | | |  |
| 1. When you made your first appointment, how long did you have to wait for a provider to see your child? | ………………….. day(s) | | |  |
| 1. On the day of your rehabilitation service appointment, how long (in minutes) do you have to wait in the rehabilitation center before you are seen by a provider? | …………….. minutes | | |  |
| 1. Do/did you pay to get the rehabilitation services? | 🗌 Yes |  | 🗌 No | 🗌 Uncertain |
| 1. If yes, please specify the amount | .................................. /taka | | | |
| 1. Do/did you need to pay any indirect cost in getting the rehabilitation center (e.g., cost of transportation)? | 🗌 Yes |  | 🗌 No | 🗌 Uncertain |
| 1. If yes, specify amount | ................................. taka | | | |
| 1. Is the rehabilitation center accessible for persons with mobility devices? | 🗌 Yes 🗌 No 🗌 Uncertain | | | |
| 1. What is your level of satisfaction with the providers’ attitude? | 🗌 Extremely dissatisfied 🗌 Dissatisfied  🗌 Neutral 🗌 Satisfied  🗌 Extremely satisfied | | | |

4.5 Reserved seats in public transportation

| Questions | Answer options | |
| --- | --- | --- |
| 1. How far (in kilometers) is the bus stop from your place of residence? | …………………… kilometers |  |
| 1. How long (in minutes) does it take to get to the bus stop? | ………………….. minutes |  |
| 1. Do/did you pay the bus fare while travel with your child with disability? | 🗌 Yes, I pay/paid the amount similar to other passengers  🗌 Yes, but I had to pay more for an extra spot for mobility device  🗌 No, I don’t pay for transportation or pay less | |
| 1. Are public transportation accessible for persons with mobility devices? | 🗌 Yes 🗌 No 🗌 Uncertain | |
| 1. What is your level of satisfaction with the bus drivers/conductors’ attitude? | 🗌 Extremely dissatisfied 🗌 Dissatisfied  🗌 Neutral 🗌 Satisfied  🗌 Extremely satisfied | |
| 1. What is your level of satisfaction with other passengers’ attitudes? | 🗌 Extremely dissatisfied 🗌 Dissatisfied  🗌 Neutral 🗌 Satisfied  🗌 Extremely satisfied | |

Are you willing to be contacted to participate in a semi-structured interview to discuss more about your experiences?

🗌 Yes 🗌 No

If yes, would you please share your contact details ……………………………

End of survey here. Thank you so much for your time.
